# Validation of the subtle and blatant prejudice towards Bolivian immigrants scale in Argentina

**DOI:** 10.1186/s41155-023-00257-z

**Published:** 2023-06-02

**Authors:** Edgardo Etchezahar, Joaquín Ungaretti, Talía Gómez Yepes, Miguel Ángel Albalá Genol

**Affiliations:** 1 Faculty of Education, International University of Valencia, 46002 Valencia, Spain; 2 Faculty of Psychology, University of Buenos Aires — CONICET, Buenos Aires, Argentina; 3 National Scientific and Technical Research Council, 1428 Buenos Aires, Argentina

**Keywords:** Bolivian, Prejudice, Right-wing authoritarianism, Social dominance

## Abstract

Even though prejudice towards Bolivian immigrants is one of the main reasons for discrimination in Argentina, there is no valid measure to assess it. The aim of this study was to explore the psychometric properties of the subtle and blatant prejudice towards Bolivian immigrants scale. In addition, we tested correlations with right-wing authoritarianism, social dominance orientation, feelings towards Bolivian immigrants, and ideological self-placement. Data was collected through a convenience sample of 431 undergraduate students from Buenos Aires, with an age range from 18 to 45 years old (38.75% men and 61.25% women). Results showed adequate psychometric properties for the scale. Moreover, significant correlations between subtle and blatant prejudice and the other psychosocial variables tested were found. Implications of these findings are discussed.

## Introduction

From the beginning of the nineteenth century, migratory movements to Argentina were key elements in the foundation of the nation and its subsequent development (Domenech & Pereira, 2017). Towards the end of the nineteenth century, the total population of Argentina amounted to 670,000 inhabitants, and, according to the National Institute against Discrimination, Xenophobia and Racism (onwards INADI, 2016), the immigrant population represented a third of the total, being most of them originally from Spain and Italy. However, due to the economic crises that Argentina went through and the improvement in the quality of life in countries like Italy and Spain, the European immigration began to descend systematically until it was replaced by immigration coming from bordering countries. Nowadays, more than 50% of the total immigrant population in Argentina comes from bordering countries (Vacotti, 2017).

Besides these changes in the migratory composition of Argentina, since the economic crisis of 2001, xenophobic discourses have increased considering immigrants from bordering countries as responsible for unemployment, collapse of the public services, and increase of urban insecurity (Domenech, 2015). In particular, xenophobic expressions against Bolivian immigrants are mostly due to perceived economic competition, either because of the scarce job offer or the perceived use of state resources (Valverde, 2015). Some discourses maintain that they do not pay taxes, or that jobs competition is unfair since they are willing to charge less for their work (Benencia, 2004). Moreover, even though Bolivian immigrants initially settled in bordering areas, they gradually moved towards the most important urban centers becoming more visible as a social group (Grimson, 2006). Together with the sanction of Law No. 25.871 in 2003 in Argentina — which considers migration as a right — and the implementation of regularization programs between 2006 and 2015, Bolivians became relevant actors in claims for the recognition of their social, cultural, and even electoral importance, demanding improvements in their neighborhoods and their full integration into the city (Rodrigo, 2021). This situation has increased the emergence of stereotyped beliefs about Bolivian immigrants turning them into victims of prejudice and discrimination (Gonzalez, 2017). In fact, 71% of Argentine citizens recognize that immigrants from bordering countries are usually targets of prejudice and discrimination, being the Bolivians the most affected since they represent the 19.1% of the total immigrants in Argentina (INADI, 2016).

### Conceptualization and evaluation of subtle and blatant prejudice

According to Pettigrew and Meertens (1995, 2001), prejudice can be divided into two broad categories: *blatant* and *subtle* prejudice. Blatant prejudice is made up of two main features: the first one is the *perception of threat and rejection* of an out-group by explaining any supposed disadvantage of that social group on the assumption that their members are genetically inferior. The second feature of blatant prejudice refers to the *opposition to intimate contact* with the out-group members by rejecting relations in which they may have more power and higher status than the in-group members (Peetigrew & Meertens, 2001).

On the other side, the authors propose that subtle prejudice is expressed through more indirect and better socially adapted ways. This kind of prejudice is composed by three subdimensions: the *defense of the in-group traditional values*, together with the idea that the out-group members do not accept them; the *exaggeration of cultural differences* between groups by using gross stereotypes; and the *denial of positive emotions* towards the members of the out-group; considering that in some cases even the non-demonstration of positive emotions towards some social groups could be a negative attitude (Passini & Morselli, 2016).

Based on the theoretical differentiation of subtle and blatant prejudice, Pettigrew and Meertens (1995) created a scale to assess both constructs. It has been used in different countries, demonstrating adequate psychometric properties for the evaluation of prejudice towards different social groups (Arcuri & Boca, 1996; Pettigrew, 1997; Ratazzi & Volpato, 2000; Espelt et al., 2006; Navas et al., 2006; Frias Navarro et al., 2009). The scale has also been translated, adapted, and validated for its use in Spanish-speaking contexts like Spain (Rueda & Navas, 1996), Chile (Cárdenas, 2006; Cárdenas et al., 2007), and Argentina (Müller et al., 2017; Ungaretti et al., 2020).

A number of key issues regarding the subtle and blatant prejudice scale arise from previous studies. For example, it has been argued if it is really possible to consider the subtle expression as a new form of prejudice (Coenders et al., 2001). Despite the fact that previous studies arrived to different factor solutions, the most frequent have been the one-factor model (global prejudice) and the two-correlated factors model (blatant and subtle prejudice) (Arancibia-Martini et al., 2016). Furthermore, many studies used a short version of the subtle and blatant prejudice scale (10 items, five for each dimension) that allows to differentiate the main two dimensions (Müller et al., 2017; Passini & Morselli, 2016). Finally, previous studies have found that subtle and blatant prejudice were correlated with other variables such as negative feelings towards the out-group members, beliefs about their rights, gender, age, social economic level, and political self-placement (Meertens & Pettigrew, 1997; Pettigrew & Meertens, 1995; Rueda & Navas, 1996).

### Relations between prejudice, ideological attitudes, feelings, and rights

Within social psychology, right-wing authoritarianism and social dominance orientation have been important psychosocial variables related to different forms of prejudice (Sibley & Duckitt, 2013). Altmeyer (1981), defined *right-wing authoritarianism* (henceforth RWA) as the covariation of three attitudinal clusters: *authoritarian submission*, *authoritarian aggression*, and *conventionalism*. Regardless of the factorial structure of the scale, the links between authoritarianism and prejudice have been identified in a large number of studies (e.g., Altmeyer, 1981; 1998; Duckitt & Sibley, 2007; Duriez & Van Hiel, 2002, Ekehammar et al., 2004; Heaven & St. Quintin, 2003; Pettigrew, 1958; Rattazzi et al., 2007; Sibley & Duckitt, 2008, 2013). Furthermore, it has been demonstrated that authoritarian individuals have higher levels of prejudice towards out-groups perceived as dangerous and threatening to the rules, values, and traditional ways of living of the in-group (Altemeyer, 1998; Duckitt & Sibley, 2007; Duckitt & Sibley, 2010).

Besides RWA, many studies have found that *social dominance orientation* (henceforth SDO) is also related to different forms of prejudice (Duckitt & Sibley, 2017; Passini & Morselli, 2016; Pelletier-Dumas et al., 2017; Duckitt & Sibley, 2010). SDO was defined as a general tendency to maintain hierarchical social relations rather than egalitarian ones (Pratto, Sidanius, Swalthworth & Malle, 1994). To assess SDO, Pratto et al. (1994) developed the social dominance orientation scale which is an excellent predictor of prejudice towards *defiant* groups since they threat the maintenance of social inequality (e.g., immigrants) (Duckitt, 2006; Duckitt & Sibley, 2007; Frey & Meier, 2004). More recent research on the field indicate that while authoritarianism has an indirect positive effect in subtle prejudice, social dominance orientation do so with blatant prejudice (Birdir et al., 2022; Brubacher et al., 2022; Passini, 2017; Ungaretti et al., 2020).

### Background in the study of subtle and blatant prejudice towards Bolivian immigrants

Previous studies on subtle and blatant prejudice towards Bolivians were developed in Latin America. However, short versions of the scale have often been used, sometimes not guaranteeing the measurement of the constructs in a broad way. In addition, despite sharing a language with other countries in which the scale has been validated, it is necessary to adapt and corroborate the suitability of the scales in the specific places where the study sample comes from. For example, in Chile, Cardenas et al. (2007) studied a sample of 324 adolescents and young people in order to explore the psychometric properties of the subtle and blatant prejudice scale (Pettigrew & Meertens, 1995). They found that after running an exploratory factor analysis (EFA), two factors emerged: *blatant prejudice* (*α* = 0.73) and *subtle prejudice* (*α* = 0.65). Years later, using data from a probabilistic survey with a sample of 896 subjects also from Chile, Cárdenas (2010) used the Pettigrew and Meertens (1995) subtle and blatant prejudice scale in order to compare the one factor model (global prejudice) and the two-correlated factor model (subtle and blatant prejudice) through EFA and confirmatory factorial analysis (CFA). The results indicated that the two-correlated factor model had better psychometric properties than the one factor model (*α* = 0.82 for subtle and *α* = 0.76 for blatant prejudice). Finally, more recent evidence (Arancibia-Martini et al., 2016) coming from a review of Cárdenas (2010) study suggested that the internal consistency for the overall scale (*α* = 0.81) and for the two dimensions (blatant *α* = 0.67; subtle *α* = 0.71) was adequate. However, because of the strong correlations between subtle and blatant sub-scales (*r* = 60, *p* < 0.01), they concluded that the one-factor model solution was the most adequate. All these studies (Arancibia-Martini et al., 2016; Cárdenas, 2010; Cárdenas et al., 2007) arrived to some key findings: people scored higher in the subtle rather than in the blatant prejudice sub-scale, women scored significantly higher than men in the blatant sub-scale, left-wing individuals scored lower than the center or right-wing ones in the blatant sub-scale, and young people showed significantly lower levels than older people in both subtle and blatant sub-scales.

Finally, following Pettigrew and Meertens (1995) classification, all the studies developed in Chile (Arancibia-Martini et al., 2016; Cárdenas, 2010; Cárdenas et al., 2007) grouped participants into three categories: *egalitarians* (low scores in both subtle and blatant prejudice), *subtles* (high scores in subtle and low scores in blatant prejudice), and *bigots* (high scores in both forms of prejudice). They asked the participants what kind of actions do the government should have taken with Bolivian immigrants’ rights, and they found that egalitarians wanted to enlarge immigrants’ rights, subtles oscillated between restrict their rights or leave them as they are, and bigots agreed with restricting them. Besides, when testing the differences between the three categories and the emotions towards Bolivian immigrants, significant statistical differences were found between bigots and both subtle and egalitarian individuals (Arancibia-Martini et al., 2016; Cárdenas, 2010; Cárdenas et al., 2007).

The aim of this study was to adapt and validate the subtle and blatant prejudice towards Bolivian immigrants scale in Argentina. In addition, we explored the correlations between subtle and blatant prejudice, right-wing authoritarianism, social dominance orientation, feelings towards Bolivian immigrants, and ideological self-placement.

## Method

### Participants

A total of 431 all first- and second-year undergraduate students from the psychology program at a large public university in Buenos Aires City were recruited for this study, with an age range from 18 to 45 years old (*M* = 24.7; *SD* = 2.18). From the entire sample, 38.8% were men (*n* = 167) and 61.2% women (*n* = 264). Regarding participant’s ideological political self-placement, 4.2% choose right, 7.4% center-right, 52.6% center, 25.4% center-left, and 7.4% left.

### Measures

The data was collected through a self-report questionnaire that included multiple scales in order to assess the following variables:

#### Subtle and blatant prejudice towards Bolivian immigrants

The 10-item subtle and blatant prejudice scale (5 for blatant prejudice and 5 for subtle prejudice) based on the originally scale developed by Pettigrew and Meertens (1995; *α* = 0.85) was adapted and validated for the purposes of this study. Responses were measured on a 5-point Likert-type scale with anchors at *1* = *totally disagree* and *5* = *totally agree*. The internal consistency levels for the subtle and blatant subscales in the present study were the same (*α* = 0.70).

#### Social dominance orientation (SDO)

The scale used was an adaptation and validation from the original scale (Pratto et al., 1994; Sidanius & Pratto, 1999) to the Argentinian context (Etchezahar et al., 2014). The ten items that composed the scale allow to distinguish between two dimensions of the construct: group dominance (e.g., “To go on in life, sometimes is necessary to pass through other groups of people,” “All the superior groups should dominate the inferior groups”) and opposition to equality (e.g., “There would be less troubles if we treated different groups in a more egalitarian way,” “Social equality should be increased”). The psychometric properties of the scale were studied in a sample of university students from Buenos Aires (*N* = 302), being both internal consistency (*α* = 0.82) and construct validity (*CFI* = 0.94; *RMSEA* = 0.07) adequate. In our study, we have observed an adequate internal consistency (*α* = 0.91). Responses were measured on a 5-point Likert-type scale with anchors at *1* = *totally disagree* and *5* = *totally agree*. Higher levels suggest a higher social dominance orientation.

#### Right-wing authoritarianism

A local version of the RWA scale (*right-wing authoritarianism*; Altemeyer, 2006) was used (Etchezahar, 2014) composed by six items. We used the unidimensional model of RWA which includes the three dimensions of the construct: authoritarian aggression (“There’s a lot of extremist and immoral people trying to ruin things; society must stop them”), authoritarian submission (e.g., “Our country needs a powerful leader able to face the extremists and immoral that nowadays prevail in our society”), and conventionalism (e.g., “Homosexuals and feminists should be praised because of their brave to challenge traditional family values,” “Nobody should follow the traditions, people should free themselves and prove different ideas and experiences”). The internal consistency of the scale (0.75 < *α* < 0.81) and its construct validity (0.97 < *CFI* < 0.98; 0.03 < *RMSEA* < 0.05) was adequate (Etchezahar, 2014). The items were measured using a 5-point Likert-type scale (1 = *totally disagree* to 5 = *totally agree*).

#### Bolivian immigrants’ rights

Following previous studies in the field (Cárdenas et al., 2007; Cea D’Ancona, 2002; Rueda & Navas, 1996), we asked “Do you think the rights of Bolivian immigrants should be….” Participants were asked to choose one of four possible answers: “expanded,” “remained the same,” restricted,” and “eliminated.”

#### Feelings towards Bolivian immigrants

To assess this variable, the question proposed by Cárdenas (2006) was adapted by asking the participants what kind of feelings awoke in them Bolivian immigrants, being the answers “very positive,” “positive,” “neutral,” “negatives,” and “very negative.”

#### Social demographic variables

An ad hoc questionnaire was developed to collect this information. The variables assessed were gender, age, and ideological self-positioning.

### Procedure

The subjects were invited to participate in the investigation voluntarily, requesting their informed consent. They were also informed that the data derived from this research would be used only for scientific-academic purposes and protected by the National Law 25.326. All participants were residents of Buenos Aires at the time the data were collected, and they were recruited for this study. In all cases, we have worked with complete questionnaires, without missing data. To validate the subtle and blatant prejudice scale towards Bolivian immigrants to the Argentine context, international methodological standards were followed as suggested by the International Test Commission (ITC) for a right adjustment of an instrument from one language context to another (Muñiz et al., 2013). Moreover, the necessary permission for the use of the scales was obtained, and procedures used by other Hispanic-speaking versions previously adapted were considered (Cárdenas, 2010; Cárdenas et al., 2007; Del Castillo et al., 2003; Müller et al., 2017). In the initial stage of the process, items were written and later depurated until arriving to a preliminary version of the scale. Subsequently, these items were analyzed by three expert judges and then administrated to a pilot sample composed by 22 participants. Finally, as a result of these procedures, many items were reformulated, and others suppressed until arriving to the 10-item final scale. These steps allowed the idiomatic adjustment of the instrument and were useful to identify the most representative items for assessing subtle and blatant prejudice constructs as the authors of the original scale suggested (Pettigrew & Meertens, 1995).

## Results

### Analysis of the subtle and blatant prejudice scale towards Bolivian immigrants

Table 1 presents the items that composed the subtle and blatant prejudice scale towards Bolivian immigrants, as well as their mean (*M*), standard deviation (*SD*), asymmetry (*S*), and kurtosis (*K*).

**Table 1:** Descriptive statistical analysis of the items and internal consistency of the subtle and blatant prejudice scale towards Bolivian immigrants in Argentina

	*M*	*SD*	*S*	*K*
BP1: Argentinians and Bolivians cannot feel comfortable between each other, even if they are friends	1.74	1.19	1.47	0.96
BP2: Bolivian immigrants occupied the jobs that should be for Argentinians	2.24	1.32	0.63	− 0.89
BP3: *Bolivian immigrants are as honest and reliable as Argentinians*	3.86	1.18	− 0.66	− 48
BP4: Most of the Bolivian immigrants that received some kind of social or economical help do not need it and could live without it if they wanted to	2.38	1.19	0.42	0.96
BP5: *I would not care if a Bolivian immigrant in a similar economic situation like mine get married with someone of my family*	4.15	1.17	− 1.19	0.39
SP1: Bolivian immigrants that live in our country and teach their children values and customs different from the ones needed to be successful in this society	2.54	1.22	0.16	− 0.88
SP2: Bolivian immigrants differ a lot from Argentinians in their beliefs and religious practices	3.10	1.10	− 0.26	− 0.35
SP3: Bolivian immigrants are very different from Argentinians in the way they teach their children to follow rules	2.87	1.18	− .08	− 0.66
SP4: Bolivian immigrants are very different from Argentinians in their sexual practices	2.43	1.01	− 0.24	− 0.55
SP5: Bolivian immigrants are very different from Argentinians in their ways of talking and communicating with others	3.08	1.24	− 0.23	− 0.94

In italic are presented the reverse items; *BP*, blatant prejudice; *SP*, subtle prejudice

### Exploratory and confirmatory factor analysis of the subtle and blatant prejudice scale towards Bolivians

**Fig. 1:**
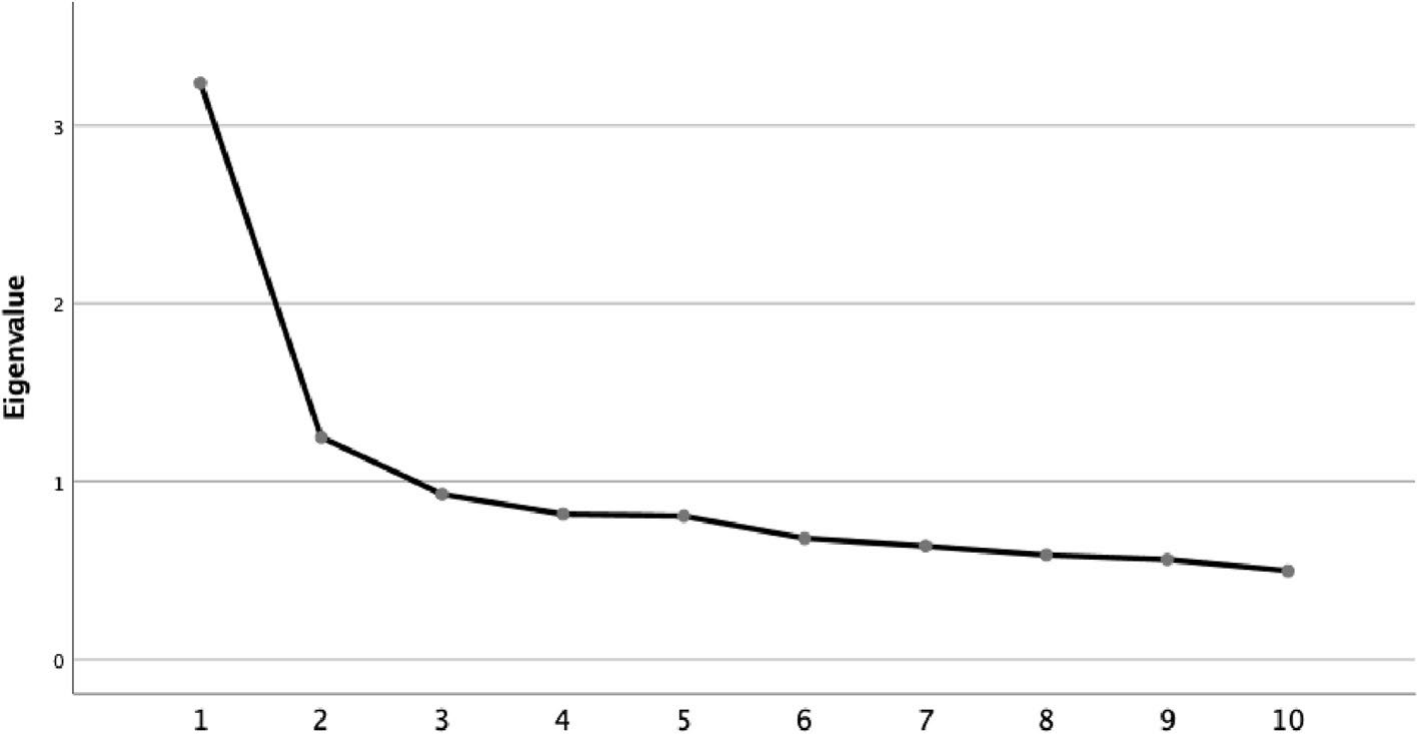
Scree plot shows that two components had eigenvalues higher than 1

A exploratory factor analysis (henceforth EFA) was conducted (Auerswald & Moshagen, 2019). These analyses were possible because of the adequate results obtained in the Keiser-Mayer-Olkin (*KMO* = 0.860) test and Bartlett sphericity test (*p* < 0.001). The sedimentation graphic showed the presence of two factors with eigenvalues > 1 (Fig. 1) (Cattel et al., 1966), five for each dimension (Table 2).

**Table 2:** Total-item correlation, Cronbach’s alpha if item deleted and rotated component matrix of the subtle and blatant prejudice towards Bolivian immigrants

*Subtle prejudice* (*α* = 0.70)	*rjx*	*α-x*	1	2
SP3: Bolivian immigrants are very different from Argentinians in the way they teach their children to follow rules	0.45	0.65	**0.78**	.05
SP2: Bolivian immigrants differ a lot from Argentinians in their beliefs and religious practices	0.46	0.65	**0.65**	.07
SP5: Bolivian immigrants are very different from Argentinians in their ways of talking and communicating with others	0.49	0.64	**0.65**	.09
SP4: Bolivian immigrants are very different from Argentinians in their sexual practices	0.49	0.64	**0.62**	0.13
SP1: Bolivian immigrants that live in our country and teach their children values and customs different from the ones needed to be successful in this society	0.44	0.66	**0.58**	0.29
*Blatant prejudice* (*α* = 0.70)				
BP4: Most of the Bolivian immigrants that received some kind of social or economical help do not need it and could live without it if they wanted to	0.43	0.67	.06	**0.76**
BP5: *I would not care if a Bolivian immigrant in a similar economic situation like mine get married with someone of my family*	0.42	0.67	− .01	**0.70**
BP3:* Bolivian immigrants are as honest and reliable as Argentinians*	0.57	0.60	0.21	**0.66**
BP1: Argentinians and Bolivians cannot feel comfortable between each other, even if they are friends	0.43	0.66	0.24	**0.61**
BP2: Bolivian immigrants occupied the jobs that should be for Argentinians	0.44	0.66	0.30	**0.59**

Note. In bold are highlighted the factorial charges according to the factor that gather them; in italic are presented the reverse items

According to the EFA, items were gathered in two factors with a total variance explained of 47.36%, being 23.69% for the subtle prejudice dimension and a 23.67% for the blatant prejudice dimension. Taking into account Coenders et al. (2001) arguments regarding subtle prejudice as a “new form of prejudice” or if it is just an expression of a global traditional prejudice, we contrasted the one-dimension model of prejudice with the two correlated dimensions model of subtle and blatant prejudice (Table 3).

**Table 3:** Comparison between the models of one and two dimensions of the subtle and blatant prejudice towards Bolivians

	*X*^2^_(gl)_	*X*^2^/_gl_	*CFI*	*AGFI*	*RMSEA*
SBPB (one dimension)	213.854_*(35)*_	6.11	0.75	0.81	0.115 [0.110–0.130]
SBPB (two dimensions)	90.140_*(34)*_	2.65	0.92	0.93	.065 [.049–.082]

SBPB (one dimension): One-dimensional model of the subtle and blatant prejudice scale towards

Bolivians; SBPB (two dimensions): Two correlated dimensions model of the subtle and blatant Prejudice scale towards Bolivians. Adequate values: *χ*^2^/_gl_ ≤ 5; CFI, *AGFI* ≥ 0.90; *RMSEA* ≤ .08

The results in Table 3 indicated a greater adjustment for the model of two dimensions. Also, it can be observed that all the total-item correlations presented in Table 2 were adequate (0.42 < *r* < 0.57), and so, the Cronbach alpha if item deleted (Hair et al., 2006). The correlation between factors was *r* = 0.38 (*p* < 0.001).

### Levels of subtle and blatant prejudice towards Bolivians immigrants according to the participant’s gender

It has studied the difference between the subtle and blatant forms of prejudice according to the participant’s gender. First, we tested the gender invariance of the scale, and all models (configural, metric, and scalar) had an adequate fit. Following the criterion of *Δ*CFI ≤ 0.01 for the strict invariance (Cheung & Rensvold, 2002), measurement invariance across gender was found. Secondly, we developed a mean comparison through *t*-statistic, identifying only significant statistically differences on the subtle prejudice scale (*t*_(421)_ =  − 2.221; *p* < 0.05; Cohen’s *d* = 0.234), being women (*M* = 2.49; *SD* = 0.97) who scored higher than men (*M* = 2.25; *SD* = 1.06).

### Judgments about the rights and feelings towards Bolivians immigrants according to prejudice typologies

In order to analyze the judgments about Bolivians’ rights and the feelings towards Bolivian immigrants, we constructed a typology based on Pettigrew and Meertens (1995) recommendations on the basis of the scores obtained by the participants on the subtle and blatant prejudice scale. Following previous studies, participants were divided in four groups depending on their high or low scores in both scales (Cárdenas, 2010; Cárdenas et al., 2007; Meertens & Pettigrew, 1997; Pettigrew & Meertens, 1995). Table 4 informs the frequencies distribution for each category (egalitarian, subtle, and bigot), and the frequencies for the category called “error,” which includes people with high scores on the blatant prejudice dimension and low on the subtle dimension.

**Table 4:** Frequencies for the different typologies of prejudice towards Bolivian immigrants

	*f*	*(%)*
Egalitarians	181	46.8
Bigots	27	7
Subtles	173	44.7
Error	6	1.5
Total	387	100

The results on Table 4 indicate that the higher percentage of subjects could be categorized as *egalitarian* (46.8%), followed by *subtle* (44.7%), *bigot* (7%), and *error* (1.5%) categories.

Consequently, this typology was used as a grouping factor to perform one-way ANOVA analysis with the other variables assessed to test the validity of the scale. We proceeded to compare the means for the variable f*eelings towards Bolivian immigrants*, perceiving statistically mean differences (*F*_(2, 431)_ = 13.309; *p* < 0.001). According to the post hoc Tukey b contrast, two groups were identified: *Egalitarians* (*n* = 180; *M* = 2.58) and *subtles* (*n* = 172; *M* = 2.85) on the one hand and *bigots* on the other (*n* = 27; *M* = 3.52). These results indicate that subtles and egalitarians negative feelings towards Bolivian immigrants were lower than those of bigots. Besides, regarding the participants’ answers about *Bolivian immigrants’ rights*, percentages are shown on Table 5.

**Table 5:** Percentages distribution according to typologies for the variable Bolivian immigrants’ rights

	Typology		
	Egalitarians	Subtles	Bigots
Expand	58.2%	43.6%	11.5%
Leave as they are	37.6%	45.4%	50.0%
Be reduced	3.0%	8.6%	23.1%
Be eliminated	1.2%	2.5%	15.4%
Total	100%	100%	100%

The question that was made to the participants was as follows: “Do you think the rights of Bolivian immigrants should be…”

According to Table 5, egalitarian participants supported the expansion of Bolivian immigrants’ rights (58.2%), while subtles vary from leaving their rights as they are (45.4%) to expand them (43.6%). In the case of bigots, 50% of the participants would leave Bolivians’ rights as they are, the 23.1% would reduce them, and the 15.4% would eliminate them. Also, there were statically differences between groups (*χ* ^2^_(2)_ = 35.535; *p* < 0.001).

### Relations between the RWA, SDO, subtle and blatant prejudice towards Bolivian immigrants

After testing the psychometric properties of the subtle and blatant prejudice scale towards Bolivian immigrants, we analyzed the correlations of both types of prejudice expressions with RWA, SDO, ideological self-placement (PI), and feelings towards Bolivian immigrants (Table 6).

**Table 6:** Relations between subtle and blatant prejudice with other psychosocial variables

	1	2	3	4	5	6
1. Subtle prejudice	0.70					
2. Blatant prejudice	0.383**	0.70				
3. RWA	0.402**	0.391**	0.82			
4. SDO	0.297**	0.394**	0.354**	0.91		
5. Feelings towards Bolivian immigrants	0.217**	0.288**	0.231**	0.133**	-	
6. PI	− 0.289**	− 0.212**	− 0.431**	− 0.279**	− 0.192**	-

*PI:* Political ideology self-placement. **. p < 0.001

As it can be observed on Table 6, all the variables were significantly correlated with subtle and blatant prejudice towards Bolivian immigrants. Likewise, as suggested by previous studies (Birdir et al., 2022; Brubacher et al., 2022; Ungaretti et al., 2020), the contribution of RWA and SDO in both forms of prejudice was analyzed. For blatant prejudice, a *R* ^2^ = 0.246 was observed, and the contribution of SDO (*β* = 0.299; *p* < 0.001) and RWA (*β* = 0.304 *p* < 0.001) was similar. However, with subtle prejudice (*R* ^2^ = 0.192), the RWA makes a greater contribution (*β* = 0.373; *p* < 0.001) than SDO (*β* = 0.139; *p* < 0.001).

## Discussion

After analyzing the psychometric properties of the subtle and blatant prejudice scale towards Bolivian immigrants in Argentina, the factorial structure identified was coherent with previous studies (Passini & Morselli, 2016). It has two correlated factors explaining the 47.36% of the total variance. As it was mentioned before, most of the critics regarding the original theoretical structure of the scale has been motivated by the high correlations (0.48 < *r* < 0.73) between the blatant and subtle subscales found in previous studies (Meertens & Pettigrew, 1997; Pettigrew & Meertens, 1995; Rueda & Navas, 1996). In this study, correlations between subtle and blatant prejudice towards Bolivian immigrants in Argentina were lower (*r* = 0.38).

After grouping participants according to their subtle and blatant prejudice levels, most of them were classified as egalitarians — lower in both types of prejudice — compared with those on Chilean studies (Arancibia-Martini et al., 2016; Cárdenas et al., 2007). Regarding the subtle typology, similar percentages than those in Chile (Arancibia-Martini et al., 2016; Cárdenas et al., 2007) were found in our sample. These results indicate some differences between Argentina and Chile, maybe related to differential measures carried out to reduce prejudice towards Bolivian immigrants. However, this results could be related to the differences between the samples studied in each country. However, prejudice towards Bolivian immigrants in Argentina has not disappeared (INADI, 2016); by the contrary, it seems to have changed from blatant towards subtler, indirect, and more socially accepted expressions of negative attitudes.

As well as results obtained by Cárdenas et al. (2007), in the present study, there were no differences between subtles and egalitarians in their feelings towards Bolivian immigrants. Differences were just found between these two groups and the bigots. Additionally, according to the judgments of the Bolivian immigrant rights, as in both Chilean studies (Cárdenas, 2010; Cárdenas et al., 2007), meaning differences were not observed between subtle and egalitarian subjects but between these two groups and the bigots who supported the restriction of Bolivian immigrant’s rights. Even though just the bigots were found to support the restriction overtly, egalitarians and subtles — which together represent almost the entire sample — did not agree with the extension of Bolivian rights, and they think they should be leaved as they are. As it was mentioned before, this situation can be also an expression of newer and subtler forms of prejudice towards Bolivians in Argentina.

The results in the present study indicated, like previous studies (Pettigrew & Meertens, 1995, 2001; Ruedas & Navas, 1996), that women scored higher than men in subtle prejudice, but not in blatant prejudice. These findings differ from those in Cárdenas study (2010), who observed higher levels of blatant prejudice towards Bolivian immigrants in women than in men. The differences in both studies could be explained by the fact that in Argentina, the percentage of women that comes from Bolivia is similar to that of men (INDEC, 2010), while in the Chilean context, women immigration far exceeds that of men (Cárdenas, 2010). This is why Chilean women may feel more threatened by the huge presence of this social group.

### Subtle and blatant prejudice, authoritarianism, dominance, and feelings towards Bolivian immigrants

As well as Passini (2017) proposed about authoritarianism and dominance having differential and indirect effects in the subtle and blatant prejudice levels, the present study found so with prejudice towards Bolivian immigrants. Along these lines, other authors (Birdir et al., 2022; Brubacher et al., 2022; Ungaretti et al., 2020) have pointed out that depending on the context, it may be RWA or SDO that makes a differential contribution to the type of prejudice, as observed in our results, since SDO would account for a differential contribution, while RWA would not. Moreover, as Brandt (2017) suggested, we also found evidence that those effects are also correlated with an individual’s political and ideological self-placement. In other words, given the expansion of Bolivian ethnic visibility in Argentinian urban centers (Grimson, 2006) and their representation linked to the insecurity and criminality, it is possible that this entails a higher level of dominance (Gonzalez, 2017).

Also, it was observed that social dominance orientation explained partly the blatant prejudice towards Bolivian immigrants. This would indicate that, for people with higher levels of blatant prejudice, Bolivians would be perceived as a defiant social group that threats the sustaining of social inequality. As previously mentioned, both the perceived economic competition because of the scarce job offer (Valverde, 2015) and the perceived use of state resources (Benencia, 2004), as well as the disputes carried out to claim for their place in the community (Rodrigo, 2021), may have contributed to this findings. However, it is necessary in future studies to more consistently test the discriminant predictive validity of the scale.

One of the main limitations of this study was related to the convenience sample (Hernández Sampieri et al., 2014) used in this paper. It does not allow the generalization of the results presented to the total population, since the random premise in the sample selection has not been met. Besides, no information was collected about participant’s immigrant background. Results from previous research suggest that reduction of social inequality (Frey & Meier, 2004) and prejudice reduction (Duckitt & Sibley, 2010) may depend on institutional and environmental conditions and on the possibility of relating between individuals. Thus, an exclusively student sample could be biased, presenting a lower level of prejudice than in a more heterogeneous sample. On the other hand, the difference in the results between some students and others could be due, as previous studies point out, to the fact that students select different disciplines based on differences in their prosocial preferences and relationships (Konow, 2019), which could influence the low number of bigots.

From what was exposed here, it is necessary to continue assessing the variables proposed with samples that include participants from other social clusters and participants having direct contact with Bolivian immigrants or at least to include questions about immigrant background in order to know if their parents were both born outside Argentina —first-generation immigrants — or in Argentina — second-generation immigrants. Finally, we are aware that the hierarchical test performed in this study is the minimum requirement to compare a single factor with a two factor model of subtle and blatant prejudice. However, since the original scale has been adapted to assess prejudice towards Bolivian immigrants, it is important to consider that items are based on the original instrument, but the results cannot fully represent results as if the original instrument was administered.

Despite these limitations, to have a version of the original scale adapted and validated for the analysis of subtle and blatant prejudice towards a social group highly vulnerable in our context and analyzing its psychological basis can contribute to develop future studies that allow the reduction of prejudice and discrimination towards that collective.

## Data Availability

The datasets used and/or analyzed during the current study are available from the corresponding author on reasonable request.
